# CoV2K model, a comprehensive representation of *SARS-CoV-2* knowledge and data interplay

**DOI:** 10.1038/s41597-022-01348-9

**Published:** 2022-06-01

**Authors:** Tommaso Alfonsi, Ruba Al Khalaf, Stefano Ceri, Anna Bernasconi

**Affiliations:** Politecnico di Milano, Dipartimento di Elettronica, Informazione e Bioingegneria, 20133 Milano, Italy

**Keywords:** Data integration, Databases, SARS-CoV-2

## Abstract

Since the outbreak of the COVID-19 pandemic, many research organizations have studied the genome of the SARS-CoV-2 virus; a body of public resources have been published for monitoring its evolution. While we experience an unprecedented richness of information in this domain, we also ascertained the presence of several information quality issues. We hereby propose CoV2K, an abstract model for explaining SARS-CoV-2-related concepts and interactions, focusing on viral mutations, their co-occurrence within variants, and their effects. CoV2K provides a clear and concise route map for understanding different connected types of information related to the virus; it thus drives a process of data and knowledge integration that aggregates information from several current resources, harmonizing their content and overcoming incompleteness and inconsistency issues. CoV2K is available for exploration as a graph that can be queried through a RESTful API addressing single entities or paths through their relationships. Practical use cases demonstrate its application to current knowledge inquiries.

## Introduction

### Background

Thanks to the continuous and massive process of deposition of SARS-CoV-2 sequences to public databases^1^, viral evolution is constantly monitored by international organizations. A huge body of research studies links the spreading of mutational processes to the virus ability to organize within more transmissible variants or to escape vaccines or treatments, thus affecting the evolution of COVID-19 disease. The process is of enormous importance for understanding the course of the pandemic; however, it is hampered by a lack of (1) coordination of terms and concepts being used, (2) methods to handle their heterogeneity and evolution in time, (3) an instrument that allows to connect concepts.

### Information quality issues

**Fig. 1:**
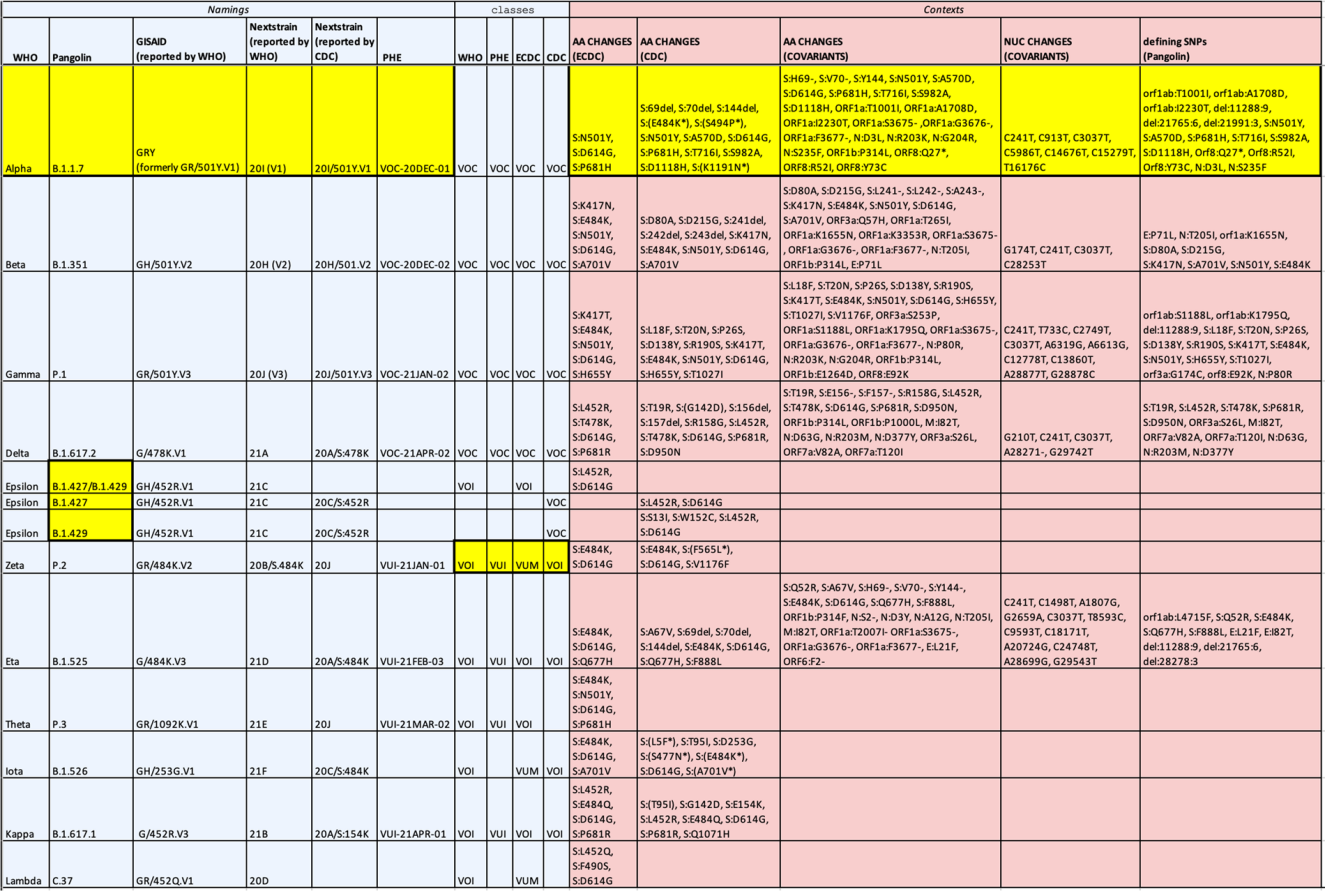
Different *Namings*, classes, and *Contexts* (i.e., characterizing mutations) given to the most known WHO-named variants available on June 18th, 2021. Information heterogeneity is highlighted in yellow; sources report different groupings, classes, names, and mutation characterizations.

Nowadays several organizations in the domain provide different descriptions of the same concepts. Figure 1 captures a snapshot of the information collected on June 18th, 2021 from such sources. This example is illustrative of both heterogeneity among descriptions and inconsistencies, all highlighted in yellow:

1. *Terminology issues*. Names are assigned by WHO^2^ (using Greek letters), but they are also provided by Pangolin^3^ as *lineages*, GISAID^4^ and Nextstrain^5^ as *clades*, or by Public Health England (PHE^6^) based on the variant importance for genomic surveillance. Nextstrain names are in turn reported by portals of both WHO and the Centers for Disease Control and Prevention (CDC^7^), with different notations for many lineages/variants (e.g., Alpha is mapped to 20I(V1) and to 20I/501Y.V1); GISAID also provides heterogeneous names (e.g., Alpha is currently assigned to GRY; formerly it was assigned to GR/501Y.V1). Mismatches may depend on updates misalignment or to partial wordings. At that time, WHO was reporting the most updated Nextstrain names^8^ whereas the CDC names were outdated.
2. *Variant identity issues*. Some sources group together multiple lineages (e.g., B.1.427/B.1.429 in WHO), while others differentiate them in terms of context and effects (e.g., in CDC), or even ignore them (e.g., in PHE).
3. *Classification issues*. Variants are classified differently by their reporting sources, e.g., the Zeta (P.2) variant was reported as ‘Variant of Interest’ (changed to ‘Alert for Further Monitoring’ two weeks later) by WHO and CDC, as ‘Variant under Investigation’ by PHE, and as ‘Variant Under Monitoring’ by the European Center for Disease Prevention and Control (ECDC^9^) at the time the observed snapshot.
4. *Characterization issues*. Variants’ characterizations (in terms of mutations that determine their assignment to a variant) are reported along different schemes and guidelines by both authoritative sources such as WHO^2^, CDC^7^, ECDC^9^, PHE^6^, and by academic/community-driven services such as CoVariants^10^, outbreak.info^11^, and Cov-Lineages.org^12^. For the Alpha (B.1.1.7) variant, we find different contexts from different authoritative sources. ECDC and CDC only describe notable Spike changes; ECDC reports only three changes, CDC reports 13 changes (however, three of them are “detected in some sequences but not all”). CoVariants^10^ (employing the Nextstrain system) reports both amino acid changes and synonymous (nucleotide) mutations. Reported amino acid changes use different notations; for example: i) the Cov-Lineages.org Lineage Report uses ORF1ab, CoVariants mentions ORF1a and ORF1b, whereas GISAID directly uses non structural proteins NSP1–NSP16; ii) Cov-Lineages.org expresses deletions as consecutive missing nucleotides, whereas CoVariants names missing amino acids one by one. Similar differences occur in all the rows.

Note that all the four described aspects has been addressed by clustering information about variants by means of unique identifiers (e.g., the Pango lineage) and then applying entity reconciliation, building one integrated information structure that organizes different SARS-CoV-2 data silos.

### Objective

The goal of this article is to provide CoV2K, a unifying, abstract model of the information about SARS-CoV-2 in terms of entities and relationships; the model links these concepts both to knowledge sources and to massive datasets of sequence data. In addition to the illustration of the model, we also explain how the model is instantiated from some of the relevant information sources and can be explored by means of a simple API, exemplified with several prepared use cases.

## Results

### Abstract model

**Fig. 2:**
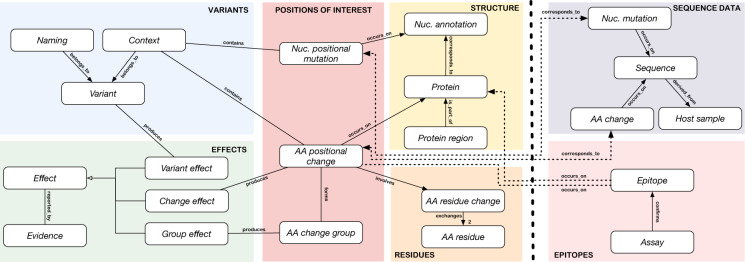
CoV2K abstract model. The areas on the left of the dashed vertical line represent knowledge about SARS-CoV-2, whereas the right areas contain its data. Within each area, entities are represented by white rectangles. Within and across areas, entities are connected by relationships, of five kinds: i) directed edges between two entities represent functional relationships, e.g., each *Context* may refer to only one *Variant*; ii) a direct edge with the marked cardinality “2” denotes a double functional relationship (one *AA residue change* involves two *AA residue*s); iii) direct edges connecting one (father) entity to many (child) entities represent a generalization hierarchy, e.g., *Effects* are either *Variant effects* or *Change effects* or *Group effects*; iv) indirect edges represent many-to-many relationships, e.g., each *Variant* may refer to many *Variant effects* and each *Variant effect* may refer to many *Variants*; v) dashed lines represent knowledge-data connections. The names of relationships are read along the direction of the arrow for functional relationships, else the direction is clear from the context.

The CoV2K abstract model, shown in Fig. 2, describes various biological aspects of viral sequences. Areas – indicated with CAPITAL letters – correspond to different facets of the abstract model. Each area contains a number of entities (rectangles, in *italics* in the text) with different attributes (not shown in the figure, typed in the text). We assume all entity names to be different and each entity to carry an identifier, with a uniquely assigned name (〈entity_name〉_id). Indirect edges denote many-to-many cardinality, binary direct edges denote one-to-many cardinality (the edge points to the entity that is functionally determined), bidirectional edges indicate one-to-one cardinality, direct edges linking one father entity to many child entities denote a generalization hierarchy. Dashed lines represent the connections between the left and right parts of the model, where the left part describes knowledge about SARS-CoV-2 and the right part describes extensive data (millions of sequences and thousands of epitopes) collected in public databases. All relationships are named except the generalization hierarchy describing effects. Next, we dedicate one section to each area, in which we provide a brief introduction of its purpose, a bulleted list – where each element corresponds to one entity of CoV2K along with its attributes – information on its connections with entities of other areas, and mention to suitable information sources for its instances. The list of all entities and attributes of the abstract model is provided in the project’s GitHub repository.

#### STRUCTURE area

SARS-CoV-2 is a single-stranded RNA virus; the two most used reference sequences, i.e., NC_045512^13^ for GenBank and hCoV-19/Wuhan/WIV04/2019^14^ for GISAID^4^ respectively present 29,903 and 29,891 bases, where each base corresponds to one of the 4 nucleotides A, C, G, or T. As in this work we chose the NCBI standard, each position in a sequence is characterized by a unique number within the 1–29,903 range. The STRUCTURE area aims to capture the information related to specific portions of the sequence.

- *Nuc. annotation* entity. The structure of SARS-CoV-2 sequences is modeled by defining a rich set of annotations, e.g., regions identified with a name with start_on_ref and stop_on_ref positions on the reference sequence.
- *Protein* entity. Aa_sequences, i.e., sequences of amino acids, form proteins; each protein maps to a specific nucleotide annotation based on its starting and ending position. Several schemes are used to denote SARS-CoV-2 protein regions; here, we adopt the scheme employed by NCBI GenBank^13^, using both polyproteins ORF1ab and ORF1a and their nonstructural proteins NSP1–NSP16 as instances of the *Protein* entity, and then mapping ORF1ab and ORF1a to NSP1–NSP16 for denoting mutations (GISAID convention). Each protein sequence has a given aa_length measured by the number of its amino acids; therefore, proteins’ positions are associated with a unique number ranging from 1 to the protein’s length.
- *Protein regions* entity. Proteins include regions with special properties having a start_on_protein and a stop_on_protein position linked to a given protein_id, as well as a describing name, a general category, and a type.

Nucleotide annotations and proteins give the positional context to nucleotide mutations and amino acid changes described next. Among possible knowledge sources, we selected NCBI Virus^15^ for proteins and nucleotide annotations and UniProtKB^16^ for extracting protein regions; we browse all SARS-CoV-2 protein-dedicated pages and include information regarding the topology and modification sites; we import all domains, repeats, regions, coiled coils, and motifs.

#### POSITIONS OF INTEREST area

Specific one base (or one residue) positions are of particular interest because they harbor mutations that can modify the behavior of the virus.

- *Nuc. positional mutation* entity. Mutations occur at specific positions of the SARS-CoV-2 nucleotide sequence, causing deletions, insertions or – most frequently – substitutions (difference encoded by the type attribute). They have a position, where the reference nucleotide is changed into an alternative, affecting a certain length of the sequence. For instance, A23403G indicates that – in the 23403rd nucleotide of the sequence – a single base of Adenine has been changed into a Guanine.
- *AA positional change* entity. Non-synonymous nucleotide mutations cause amino acid changes within specific proteins, occurring in a position where a reference residue has been changed into an alternative residue linked to a specific protein_id for a given length); these have a major influence on the protein functionalities. When type is a deletion, the alternative residue is encoded with a dash “-”. Insertions only have a position and an alternative string of arbitrary length. Amino acid changes are denoted by strings, e.g., S:D614G denotes the substitution, at the 614 position of the Spike protein, of the amino acid Aspartic Acid (D) with the amino acid Glycine (G).
- *AA change groups* entity. As several changes may jointly produce stronger effects, it is also important to group change.

Note that this is the core area of the model, linking to all other areas: positions of interest fall within given structures of the virus; they have characteristics that depend on the single residues that are changed; positions, when mutated, lead to effects on the phenotype of the virus and, when exhibited together on same viruses, form variants. In principle, all positions are susceptible to variation; any nucleotide base or amino acid residue can be changed to any of the other three or deleted, and insertions may occur at any point of the sequence and be of arbitrary lengths. However, there is only a limited number of mutations that happen in practice. Thus, the content of this area is computed based on the presence of mutations i) in variants characterizations by authoritative sources (*Context* entity), ii) with documented effects (*Effect* entity), iii) in the data entity *AA change*. Protein names and nucleotide/amino acid changes notations need normalization to enable comparisons between sources. Regular expressions and string manipulation are used for converting them into the GISAID nomenclature (for mutations) and the UniProtKB nomenclature (for protein names).

#### VARIANTS area

Using phylogenetics, viral evolution is described by trees. Each sequence is mapped to a node and is placed in the tree based on its distance to other existing nodes. The distance between any two nodes takes into account the specific nucleotide and/or amino acid changes carried by both of them. This mapping allows the partitioning of sequences into clusters, captured at different levels of the phylogenetic tree by different organizations (e.g., lineages, clades).

- *Variant* entity. The term variant is commonly used for the clusters that become predominant (highly prevalent) in given locations at given times. Note that, when variants convey modifications in the phenotype of the virus (largely verified by the scientific community), they become new strains^17^.
- *Naming* entity. Each variant carries several names (naming_id) and classes (v_class, e.g., VoI for Variant of Interest or VuM for Variant under Monitoring) assigned by different organizations (org).
- *Context* entity. The variant is associated to several nucleotide mutations and amino acid changes by different organizations or computational rules over data (we refer to this as the owner), clarified by a rule_description. As an example, variants on the ECDC source are only characterized by three to five mutations on the Spike protein.

CoVariants provides very complete characterizations of both amino acid changes and nucleotide mutations; moreover, we may have a characterization that comprises all and only those mutations that appear in at least 75% sequences that are assigned to a particular lineage (i.e., to the related variant). Such lists of mutations are part of the *Nuc. positional mutation* and *AA positional change* entities from the POSITIONS OF INTEREST area. Variants are also linked to their *Variant effects* in the EFFECTS area. In CoV2K, we consider the variants as reported in CoVariants^10^ and in PHE^6^. Based on a comprehensive search on systems currently available, these two resources proved to be the most comprehensive (for characterizations) and promptly updated (on other organization namings). Our methods extract information from the repositories connected to these websites and, for each variant, they report names from many different organizations. Such information is encoded in the specific naming schemes employed by the organization (e.g., names such as ‘S.501Y.V2’ are typical of Nextstrain, while ‘VUI-21JUN-01’ are typical of PHE).

#### RESIDUES area

Although the effects of amino acid changes significantly depend on their position on proteins, some characteristics depend just on the specific change.

- *AA residue change* entity. Each substitution in *AA positional change* in the POSITION OF INTEREST area is connected to this entity in the RESIDUES area. Each *AA residue change* involves two (see cardinality marked with 2 in the Fig. 2) residues (entity *AA residue*), respectively named as reference and alternative, and is further characterized by the grantham_distance^18^ that measures the structural difference between the two residues’ molecules and determines the type of the change (i.e., radical or conservative, being 66 the threshold distance).
- *AA residue* entity. Each residue holds given properties (i.e., molecular_weight, isoelectric_point, hydrophobicity, potential_side_chain_h_bonds, polarity, r_group_structure, charge, essentiality, side_chain_flexibility, and chemical_group_in_the_side_chain).

The mentioned properties are described using classical sources such as NCBI Structures^19^, AAindex^20^ or authoritative chemistry books^21^.

#### EFFECTS area

The phenotype of SARS-CoV-2 can be strongly affected by given amino acid changes that arise on new viruses. The most relevant effects of amino acid changes depend on their position in proteins; the most critical effects are due to changes that fall in the Spike protein, e.g., on the Receptor Binding Domain (RBD) region or in its proximity.

- *Effect* entity. It is specified by a type, referring to i) epidemiological impacts (including, e.g., viral transmission, infectivity, disease severity and fatality rate); ii) immunological impacts (including, e.g., sensitivity to monoclonal antibodies and binding affinity to hosts’ receptors – yielding to vaccine escape); iii) protein kinetics impacts (such as protein flexibility and stability); iv) treatments impact (e.g., vaccine efficacy and drug resistance). The presence of the change may yield an increase or decrease of the impact (encoded in the lv attribute); the effect is usually reported as a result of a scientific study that used a given method (epidemiological, experimental, computational or inferred). Such effects can be referred to *variant effects*, to individual *changes effects* or to changes’ *groups effects*.
- *Evidence entity*. Each effect is reported through written documents, which could be publications, preprints, or curated sources (type of evidence), characterized by citation, uri, and publisher (e.g., preprint servers such as bioRxiv or medRxiv, forums such as Virological, or any other academic literature editor).

A preliminary taxonomy of effects has been studied previously^22^, being inspired by the CIDO ontology^23^ and is constantly enriched thanks to continuous comparison with authoritative sources such as ECDC (which now informs on the transmissibility, immunity, severity of variants) and the WHO (which reports “working definitions” of variants, stating that “the understanding of the impacts of variants may fast evolve”). The complete and updated taxonomy that we employ can be inspected on the project’s GitHub repository. Effects of single or grouped changes have been extracted manually from literature (according to the process described in^22^) or retrieved from sources such as COG-UK/Mutation Explorer^24^. Variants effects are available on ECDC. Harmonization is critical for effect-related information. With respect to variation effects reported in published literature or genomic surveillance websites, in many cases, the reported effects are contrasting with each other and/or different in their significance. Indeed, the linked references may be several, some could be peer-reviewed publications but others may also be preprints; moreover, methods to derive the effects may be experimental or computational.

#### SEQUENCE DATA area

On January 13th, 2020 the first SARS-CoV-2 reference sequence was deposited to GenBank^25^; at the beginning of 2022 the database contains more than four million sequences, being the largest fully open deposition platform. In parallel, about eight hundred thousand sequences have been deposited only in the United Kingdom to COG-UK^24^. Here, we do not consider the GISAID^4^ dataset as it does not allow data redistribution. In our previous work^26^, we described a conceptual model with the following entities.

- *Sequence* entity. The viral sequence as central concept with metadata about their origin (accession_id in the source_database), their sequencing characteristics – such as length and percentages of unknown or GC bases (n_percentage and gc_percentage).
- *Host sample* entity. It describes the connected biological aspects: the host organism properties, including location (in terms of continent, country, and region), collection_date, and host_species.
- *Nuc. mutations* and *AA changes* entities. Since sequences undergo variant calling pipelines, we also represent their nucleotide-level mutations and amino acid-level changes.

These mutations are linked to the abstract model concepts of the POSITION OF INTEREST area (of which they share the schema and attributes), thus they can be associated with the regions of the STRUCTURE area and to their RESIDUES information. GenBank and COG-UK Data is imported using the ViruSurf pipelines^27^ which also provides data curation.

#### EPITOPES area

Epitopes are strings of amino acid residues from a pathogen’s protein possibly recognized by antibodies and B/T cell receptors. They can activate an immune response from the host and are thus employed in testing assays, treatments, and vaccines. Amino acid changes that fall within epitope segments may compromise their stability and thus affect immune response.

- *Epitope* entity. It models epitopes with specific epitope_start and epitope_stop positions linked to a given protein_id and are appropriate for specific host_species (typically humans or mice, but also genetically modified organisms).
- *Assay* entity. Epitopes are confirmed by assays, which may give positive or negative outcomes. Assays can be of different assay_types (i.e., B cell, T cell or MHC ligand) and mhc_classes; for T cell assays an hla_restriction is defined, restricting the population on which the epitope would be effective.

Data is imported from the Immune Epitope Database (IEDB^28^), the largest, open-source collection of epitopes; we use the extraction pipeline described in EpiSurf^29^.

### CoV2K sources integration

The CoV2K abstract model has allowed to provide practical guidance for the design of an integration pipeline that gathers different data sources and their data types within a single infrastructure. The details of such framework are given in the “Methods” section, which describes the extraction, transformation, loading, and harmonization of CoV2K data. The harmonization step includes several operations to achieve homogeneous and interoperable content of the resulting knowledge base, including 1) reconciliation of the different representations of variants; 2) removal of duplicate mutations; 3) cleaning of duplicate evidence papers; and 4) translation of mutation coordinates into a single system of reference. All operations are needed for resolving the problems of information quality described in the introduction section.

### CoV2K content exploration

We provide a simple RESTfulAPI (base URL: http://gmql.eu/cov2k/api/) that exposes one endpoint for each entity of CoV2K, e.g., for the *Evidence* entity we use the endpoint /evidences. For each endpoint, there are four possible uses:

- Without parameters (e.g., /evidences), returning all the instances of the entity.
- With a path parameter specifying the entity identifier (e.g., /evidences/XYZ), returning only the instance with the given identifier.
- With a query parameter specifying an attribute-value pair for that entity (e.g., /evidences?type = preprint), returning the set of evidences with the given type.
- With a query parameter linking that entity to another entity through a relationship (e.g., /evidences?effect_id = XYZ), returning the set of instances of the first entity that are linked to the instances of the second entity with the specified identifier.

Note that, given two entities X and Y connected by a relationship, it is possible to extract the instances of one of them connected to a given instance of the other one. Recall that entity names are unique and that entity identifiers are constructed from entity names (i.e., 〈entity_name〉_id). Users can control the production of results of queries by means of pagination parameters limit and page; the former sets the number of instances to be produced within a page of results (e.g., 100), the latter indicates the specific page to be displayed. Pagination is mandatory for the queries over data entities (e.g., *Sequence*, *AA change*…) as they may return very large results.

Note that the endpoint/effects accepts as path parameters identifiers of variants, of groups of amino acid changes, or of single amino acid changes. Moreover, given that each residue change is connected to exactly two residues, the endpoint/aa_residues invoked on a given aa_residue_change_id returns two instances corresponding to the reference and alternative residues, whereas two specific endpoints/aa_residues_ref (resp./aa_residues_alt) can be used to return only information regarding the reference (resp. alternative) residue.

The relationships of the abstract model can be combined (chained) one after the other through the /combine endpoint, e.g., /combine/evidences/effects?aa_positional_change_id=S:L452R extracts the evidences reporting effects on the Spike mutation L452R. Pagination applies to the combination result and is mandatory if the combination result refers to a data entity. A basic error handling mechanism prohibits users to build combinations with cycles (i.e., strings with repeated entities are illegal). Using path parameters that are not part of the last specified entity is also not allowed (e.g., /epitopes/variant_id = XYZ is illegal). Finally, as intermediate results of combinations may be quite large, we set a maximum threshold on their size (say 10,000 instances); when the threshold is overcome, the query fails and the user can then use shorter combinations or more restrictive query conditions.

### Use cases

#### Use case 1

**Fig. 3:**
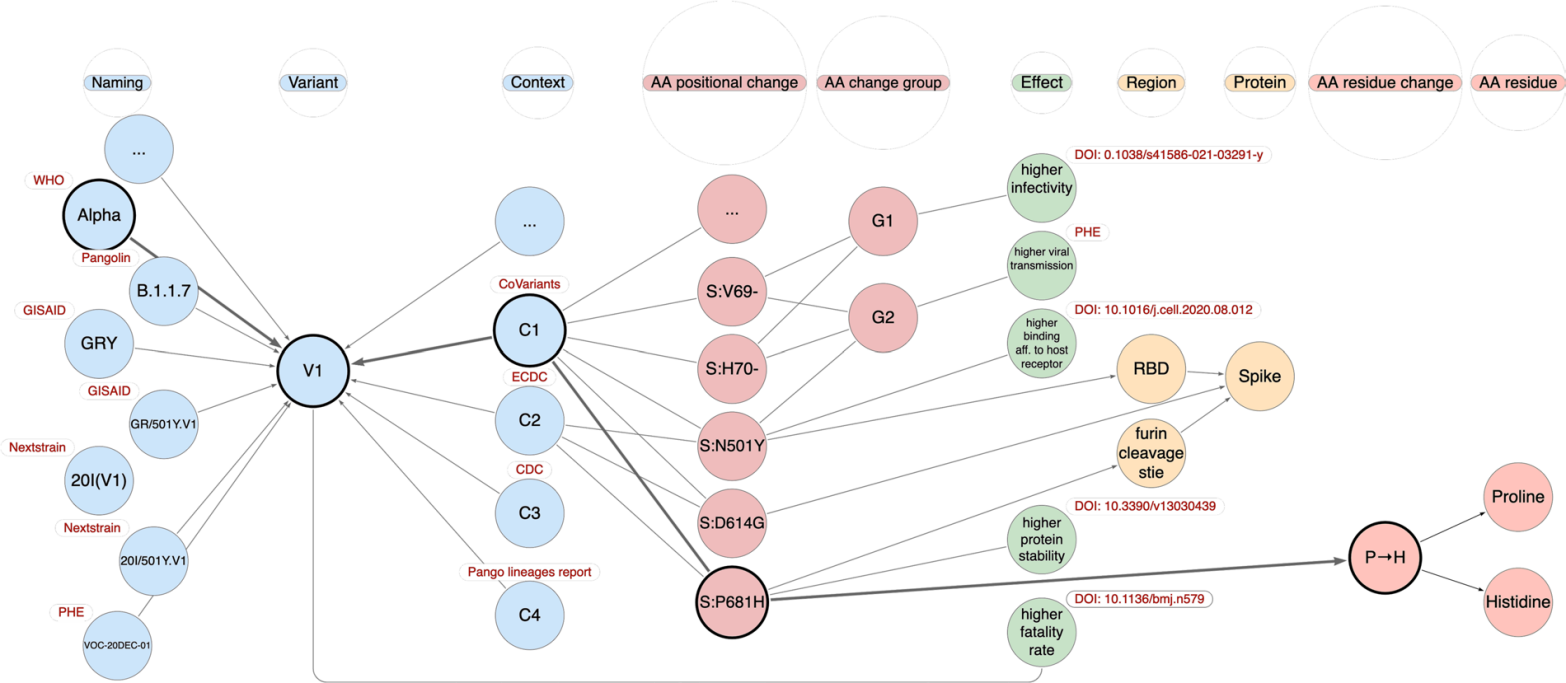
A representative instance of CoV2K, highlighting a few illustrative concepts and connections. The example refers to a variant identified as V1 and best known as Alpha (using the name assigned by the WHO organization); several alternative names are given by other organizations (red labels). The variant is associated with contexts C1–C4, each assigned by a different organization. Each context includes several amino acid positional changes. Context C2, provided by ECDC, only includes the three most representative changes on the Spike protein. Context C1 includes 24 amino acid changes – most of them are omitted in the figure. The example shows overlaps between representative amino acid changes. All changes are linked to their protein regions, possibly through their sub-regions (e.g., RBD). Effects are linked to variants, to groups of changes, or to individual changes; they are labeled with their evidence source: an organization or publication (red labels). Finally, the P-H change links to Proline and Histidine residues. Bold lines highlight one of the paths captured by the query in Use Case 1.

*What are the characteristics (Grantham distance and type) of the residue changes of the Alpha variant*? Some of the instances involved in this query are represented in Fig. 3. Let V1 be the identifier of the variant having Alpha as name (provided by the WHO organization). For evaluating its characteristic amino acid changes, we need to consider all the positional changes included in the contexts defined for V1 (in the current implementation, we include the ones defined by the owners CoVariants and Public Health England). Then, for all such positional changes, we consider the involved residue changes and extract their information, including the Grantham distance and type (radical or conservative). We compose the corresponding query as /combine/aa_residue_changes/aa_positional_changes/contexts/variants?naming_id=Alpha.

#### Use case 2

*Which amino acid changes of VOC-20DEC-02 fall within the Receptor Binding Domain (RBD)?* According to UniProtKB annotations the Receptor-binding domain falls within the 319 and 541 positions of the Spike protein. We extract the amino acid positional changes related to the requested Variant of Concern, with the following query /combine/aa_positional_changes/contexts/variants?naming_id=VOC-20DEC-02. From the result list, we select the amino acid positional changes that have: i) the same protein_id as the one of RBD (protein S); ii) a position that is included in the within the start_on_protein and stop_on_protein range of RBD.

#### Use case 3

*Which are the effects of the variants that include the Spike amino acid change D614G?* We interpret this query as the effects of variants that include D614G in at least one of their contexts. We first extract the S:D614G change, then we get all contexts that contain it; from the context we understand the related variants and, finally, we reach the effects of each of them; we use the following combined query /combine/effects/variants/contexts/aa_positional_changes/S:D614G.

#### Use case 4

*Which epitopes are impacted by amino acid changes with documented effects on the binding affinity to the host cell receptors?* As a first operation, effects of type “binding_to_host_receptor” are extracted; then amino acid positional changes connected to such effects are retrieved and their positions are intersected with the range [epitope_start,epitope_stop]. Only epitopes that include at least one of the considered changes are returned in the result. As an example, we can perform the request /combine/epitopes/aa_positional_changes/effects?type=binding_to_host_receptor&limit=100&page=1. As the combination includes a data entity (epitope), pagination parameters must be provided; follow-up queries which consider the next pages (e.g., 2,3, …) can be used to further inspect the results.

## Discussion

CoV2K is an abstract model consisting of entities and relationships; the model is sub-structured within well-identified interconnected areas, representing the facets of the information about SARS-CoV-2 virus; areas in the left represent knowledge, areas in the right represent data. CoV2K provides a query system for supporting queries that arbitrarily interconnect data and knowledge.

For what concerns knowledge, we have chosen the information sources so that they are the most updated in the landscape of SARS-CoV-2-related knowledge and they provide a tight update schedule. Moreover, our ETL and harmonization pipelines for feeding CoV2K have been designed to allow easy extension of its content by future addition of data sources when these become available and are deemed trustworthy.

For what concerns data, CoV2K includes two large databases. We previously developed the ViruSurf database^27^ (http://gmql.eu/virusurf/), which at the beginning of 2022 includes around 4 million sequences from GenBank and COG-UK with both nucleotide mutations and amino acid changes. Our pipelines reload and curate data regularly. We also include in CoV2K the Immune Epitope Database (IEDB, https://www.iedb.org/) containing about 6.5 K epitopes defined for SARS-CoV-2.

In the last year, we built a number of systems that allow to elaborate SARS-CoV-2 data and are supported by external knowledge (only covering portions of CoV2K):

- VirusViz^30^ allows to partition a sequence set of interest into groups and visualize comparatively their mutation distributions, with several options for highlighting *AA positional changes* and *Protein regions* of interest that belong to CoV2K, possibly selecting them based on their effect explained in literature evidence.
- EpiSurf^29^ allows users to analyze sequence mutations in the context of epitopes. Users may direct the mutation analysis towards specific *AA residue changes* by browsing the properties of *AA residues* and their relative Grantham distance, extracted from CoV2K.
- ViruClust^31^ is a tool for comparing SARS-CoV-2 genomic sequences and lineages in space and time not requiring any computational background to users; particularly interesting amino acid changes can be highlighted in the analysis if they are part of a *Context* of ECDC or belong to the amino acid changes of >75% of a variant. In addition, the barplots of mutations along the protein sequences allow to highlight *Protein regions* known in UniProtKB as domains, functionally characterised sites and glycosilation sites.

In all these experiences, mastering the interplay between data and knowledge in SARS-CoV-2 has proven to be extremely useful. Our current version of CoV2K system is undergoing continuous updating of information. We are designing semi-supervised methods for extracting content from the CORD-19 literature corpus^32^ to continuously collect instances of knowledge-related entities.

Many efforts aim at systematizing knowledge areas related to COVID-19. CoV2K is not competing with the COVID-19 Ontology^33^, CIDO^23^, COVID-19 Infectious Disease Ontology^34^, the COVID-19 Disease Map^35^, or the COVID-19 knowledge graph^36^, as they are essential for understanding the ontological properties of COVID-19 (mostly on the aspects of the disease), but are not aimed at linking large datasets about SARS-CoV-2 as CoV2K does. We instead propose an abstract model focused on SARS-CoV-2 sequences and their mutations/variants. CoV2K offers a tangible route map for understanding the connections between concepts and data related to SARS-CoV-2. In building the CoV2K content, we have employed a classical data integration process driven by an abstract model, with pipelines for the integration and harmonization of different data silos, and display of results by means of a flexible application programming interface. The linking of CoV2K concepts to our web resources is a step forward in promoting FAIR principles^37^, as it facilitates – at the conceptual level – the interoperability between public data sources and open knowledge and – at the practical level – the creation of several future systems that will exploit the new possibilities allowed by interlinking data and knowledge.

## Methods

### From data extraction to loading

The employed data sourcing and management methods are based on state-of-the-art ETL methods, with the following steps: i) Extract, i.e., download data from a set of sources (specified in the following) and define a parser to find the desired information within the source data structure; ii) Transform, i.e., map source data to a shared MongoDB (https://www.mongodb.com/) representation (where entities become *collections* and instances become *documents*, according to the typical terminology of NoSQL document databases); iii) Load, i.e., insert transformed collections of documents into a MongoDB instance without checks on the previous state of the database content; iv) Harmonize, i.e., normalize collections exploiting MongoDB aggregation framework to integrate and/or remove duplicate documents, homogenize attributes vocabularies and compute missing/implicit information (e.g., org of *Variant* – inferred from the naming pattern). For each area, we selected sources of information that allow to fill the content of entities and relationships. More specifically:

I). The VARIANTS area uses information from CoVariants.org^10^ and PHE^6^ for both *Naming* and *Context* entities.
II). The EFFECTS area uses information extracted from COG-UK Mutation Explorer (for *Change Effects*) in addition to our manually curated list of effects (described by Khalaf *et al*.^22^) which also includes a small number of *Group Effects* and now refers to an updated version of effects taxonomy (available on the project’s GitHub repository). *Variant Effects* are collected from the ECDC report^9^ and from CoVariants web page^10^. We do not target completeness as this is unfeasible at the moment and requires a stronger methodological setting; we are in the process of designing a semi-automatic procedure to select such mutation/variant-specific effects which will allow us to scale up the instantiation of this area.
III). The POSITIONS OF INTEREST area includes mutations that appear in the *Context* of some variant, or that are connected to *Effects*. In addition, we retrieve all the distinct mutations (of length 1) available from our ViruSurf database^27^.
IV). The STRUCTURE area includes *Nuc. annotations* and *Proteins* from the NCBI reference sequence NC_045512^13^ and *Protein regions* from UniProtKB^16^.
V). The RESIDUES area uses information retrieved from the Amino Acid Explorer of NCBI Structures^19^ in Sept. 2020 and checked against another authoritative reference^21^ as the NCBI resource has been discontinued.
VI). For the SEQUENCE DATA area, *Sequence* and *Host sample* information derives from GenBank^25^ and COG-UK^24^. *Nuc. mutations* and *AA changes* are instead computed within the internal pipelines of ViruSurf^27^.
VII). For the EPITOPES area, *Epitope* and *Assay* are filled with data derived from IEDB^28^, previously imported using our EpiSurf^29^ pipelines.

**Fig. 4:**
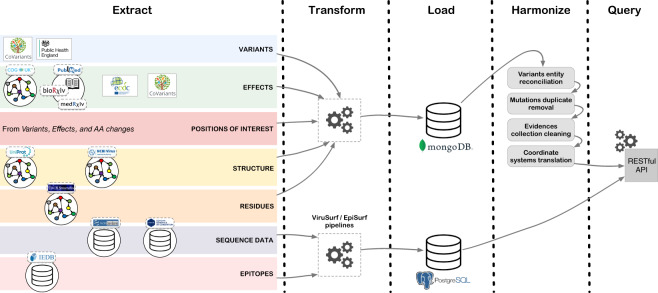
Data integration pipeline. For each area, we show the employed sources from which information is extracted, transformed and loaded, in a MongoDB instance (Knowledge areas) or a PostgreSQL instance (Data areas). A number of harmonization modules are applied to the Knowledge parts which are then ready to be queried with the RESTful API.

For the most relevant sources that we mentioned (see Fig. 4), we discuss the process that involves automatic extraction, transformation and loading modules.

#### CoVariants

We refer to the JSON at https://github.com/hodcroftlab/covariants/blob/master/web/data/clusters.json, where variants are clustered and identified with unique names assigned by Nextstrain/Covariants, enriched with a variant characterization in terms of amino acid changes and nucleotide mutations. We transform parsed data into the MongoDB schema, outputting a set of documents for the variants, aa_positional_changes and nuc_positional_mutation collections.

#### Public Health England

We use https://github.com/phe-genomics/variant_definitions/tree/main/variant_yaml (aligned to https://www.gov.uk guidelines). which contains a set of files – one for each PHE variant – describing its context (i.e., amino acid changes and nucleotide mutations) and the correspondence of this variant to other known variant names (Pango lineage, GISAID clade, etc.). We finally load a set of documents describing the variants according to PHE, including the variant characterization in terms of amino acid changes or nucleotide mutations and the name of this variant according to other organizations.

#### COG-UK Mutation Explorer

The Mutation Explorer of the COG-UK^24^ is available at http://sars2.cvr.gla.ac.uk/cog-uk/. We considered the information contained in the tabs “Antigenic Mutations” and “Drug Resistance”, reporting the effects of several hundreds of amino acid changes of the Spike protein and the articles describing such effects. We extract information from the available tables and convert it to the JSON MongoDB documents of the effects and evidence collections. As the purpose of this page is to report mutations that decrease the efficacy of specific monoclonal antibodies or treatments, we set the effect level (field lv) to “lower” by default. The effect type is understood from the “Escape mutation details” column, yielding values (such as sensitivity to neutralizing monoclonal antibodies, convalescent sera or vaccinated sera) mapped onto our effects taxonomy. When COG-UK reports multiple effect kinds for an amino acid change, multiple effects are generated in the database for that change. The column “References” provides our citation (author of the article) and the link (i.e., a DOI) for the evidence collection.

#### ECDC

We retrieve variants effects from https://www.ecdc.europa.eu/en/covid-19/variants-concern which is constantly updated by the European Union. For Variants of Concern, of Interest and Under Monitoring, we inspect the columns informing on evidence for impact on transmissibility, immunity on severity. We map such definitions on our effects taxonomy and extract the references referring to instances contained in the variants collection.

#### NCBI Virus

We refer to the reference sequence located at https://www.ncbi.nlm.nih.gov/nuccore/NC_045512. An XML structured file is analyzed and the information is used to fill the nuc_annotations and proteins collections.

#### UniProtKB

We query UniProtKB annotations by means of the EBI API (base URL: https://www.ebi.ac.uk/proteins/api/features/). We call multiple endpoints, one for each protein of interest, obtaining JSON files in output. We consider their fields Protein, Type, Category, Description and Begin-End coordinates and exclude instances of “VARIANT” and “MUTAGEN” types.

#### IEDB

We regularly download and process experimental epitopes and related assays information from the IEDB Database Export site (https://www.iedb.org/database_export_v3.php, see “CSV Metric Export” section). The epitopes attributes available in CoV2K are copied as is from the origin; each epitope is linked to the relevant assay type (combining the fields “assay_type”, “mhc_class”, and “hla_restriction”). This pipeline is embedded and shared with EpiSurf^29^.

### Data harmonization

#### Variants entity reconciliation

Variant names are reported in multiple sources in different ways; each source possibly provides also the characterization in terms of amino acid changes and nucleotide mutations. We here discuss how same real-world entities (variants) are recognized and represented in our MongoDB instance. This step is performed directly on the database collection of variants (after having loaded the data in MongoDB); it is thus independent from the imported sources and can be repeated as many times as needed. We call *clusters* the ensemble of information (related to variants’ *Naming*, *Context* and *Variant Effects*) that collectively characterizes a variant. Final variants’ clusters are produced starting from initial clusters through a sequence of operations on our MongoDB instance:

- label each input variant cluster with a unique cluster-id;
- split each cluster into items 〈*original variant name*, *variant organization*, *cluster-id*〉, where the organization is retrieved using regular expression that identify the specific known organizations naming schemes.
- isolate items without a corresponding Pango lineage (which will represent singleton independent clusters);
- group the remaining items by Pango lineage, which becomes the identifier of the final cluster, paired with a list of cluster-ids referencing the original clusters;
- in each final cluster, add the union of the properties (namings, amino acid changes, nucleotide mutations, and effects) of the referenced original clusters;
- remove duplicates from variants’ namings;
- add to the set of final clusters the original clusters without any Pango lineage.

**Fig. 5:**
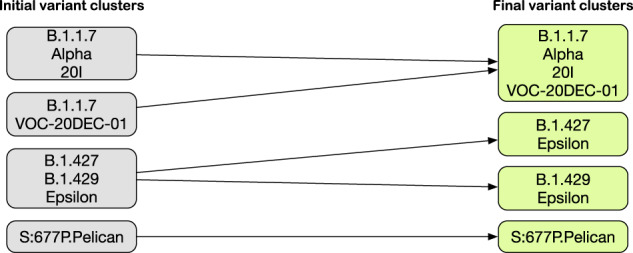
Examples of record resolution regarding the Alpha variant, the Epsilon variant, and a specific lineage spread in the United States during Summer 2020 (called Pelican in CoVariants.org).

Figure 5 shows an excerpt of the initial clusters in our database and how they are processed by the entity record reconciliation algorithm to produce final clusters. The first two clusters are recognized as referring to the same B.1.1.7 Pango lineage (used as identifier). Differently, the cluster B.1.427/B.1.429 put together two different variants that are then separated. Lastly, initial clusters that do not have a corresponding Pango lineage are kept unchanged.

#### Mutations duplicate removal

Amino acid changes and nucleotide mutations are inserted in the MongoDB instance without any check on duplicate entities. It does often happen that amino acid changes and nucleotide mutations – characterizing a variant, producing an effect, or appearing in the data entity *AA change* – are loaded multiple times. A MongoDB aggregation pipeline groups the documents of the aa_positional_changes (resp. nuc_positional_mutations) collection based on the aa_positional_changes_id (resp. nuc_positional_mutations_id) and automatically eliminates the replicates, guaranteeing that also other collections pointing to mutations reference the unique identifier.

#### Evidence collection cleaning

The ETL pipeline for COG-UK Mutation Explorer can generate many duplicate evidence entries, each of which is linked to a different effect. At the time of loading, we do not check if the effect evidence sources are already present in the DB. In MongoDB, we group these *Evidence* records (with uri, citation, publisher, and type) into a single document and reference multiple effects by their identifier.

#### Coordinate systems translation

All information in CoV2K related to positions on proteins of SARS-CoV-2 must be aligned to a same coordinate system. As discussed in the STRUCTURE area description, we employ the coordinates defined by NCBI reference sequence NC_045512^13^ and then use the GISAID/UniProtKB naming convention, with the following consequences: i) ORF1ab coordinates are translated into the corresponding coordinates of its subproteins NSP1, …, NSP16. ii) ORF6 protein becomes NS6 (non-structural protein 6, see https://www.uniprot.org/uniprot/P0DTC6). Such translations are applied to the entities *AA positional change*, *Protein*, *Protein region*, and *AA change*.

## Data Availability

CoV2K can be inspected by means of an interactive API (base URL: http://gmql.eu/cov2k/api/), with documentation of all its endpoints and links to a list of CoV2K entities/attributes and to a taxonomy of effects. Use cases are commented at http://gmql.eu/cov2k/api/usecases.

## Code availability

The code for extracting information from data sources and for the harmonization of CoV2K content is available on the project’s GitHub repository https://github.com/DEIB-GECO/cov2k_data_collector/.
